# Single-Trial Cognitive Stress Classification Using Portable Wireless Electroencephalography

**DOI:** 10.3390/s19030499

**Published:** 2019-01-25

**Authors:** Justin A. Blanco, Ann C. Vanleer, Taylor K. Calibo, Samara L. Firebaugh

**Affiliations:** 1 Electrical and Computer Engineering Department, United States Naval Academy, Annapolis, MD 21402, USA; vanleer@usna.edu (A.C.V.); tkcalibo@gmail.com (T.K.C.); firebaug@usna.edu (S.L.F.); 2 Department of Biomedical Engineering, Lund University, 221 00 Lund, Sweden

**Keywords:** Electroencephalography, Cognitive stress, Biomedical signal processing, Brain–computer interface, Stroop test

## Abstract

This work used a low-cost wireless electroencephalography (EEG) headset to quantify the human response to different cognitive stress states on a single-trial basis. We used a Stroop-type color–word interference test to elicit mild stress responses in 18 subjects while recording scalp EEG. Signals recorded from thirteen scalp locations were analyzed using an algorithm that computes the root mean square voltages in the theta (4–8 Hz), alpha (8–13 Hz), and beta (13–30 Hz) bands immediately following the initiation of Stroop stimuli; the mean of the Teager energy in each of these three bands; and the wideband EEG signal line-length and number of peaks. These computational features were extracted from the EEG signals on thirteen electrodes during each stimulus presentation and used as inputs to logistic regression, quadratic discriminant analysis, and k-nearest neighbor classifiers. Two complementary analysis methodologies indicated classification accuracies over subjects of around 80% on a balanced dataset for the logistic regression classifier when information from all electrodes was taken into account simultaneously. Additionally, we found evidence that stress responses were preferentially time-locked to stimulus presentation, and that certain electrode–feature combinations worked broadly well across subjects to distinguish stress states.

## 1. Introduction

This work examined the usefulness of scalp electroencephalography (EEG) recorded by non-medical-grade equipment for discriminating cognitive stress states in human subjects. We used a Stroop-type test [1,2] to induce stress, and then used computational EEG features in conjunction with state-of-the-practice statistical learning methods to classify stress state on a single-trial basis. The present study was an extension of our prior work [3] investigating the potential and limitations of low-cost EEG headsets for classifying cognitive stress in non-clinical settings.

An extensive literature review exploring the definition of stress was presented by Staal [4]. In this review, the scientific community’s difficulty in finding a consensus definition of the stress concept is acknowledged. Staal described the two traditional models for stress: stimulus-based and response-based. The stimulus-based model defines stress as the application of certain conditions (“stressors”) that disturb the “normal” functioning of an individual (e.g., time pressures, physical discomfort, and excessive workload). The response-based model defines stress by the pattern of responses (behavioral, cognitive, and affective) that result from exposure to a stressor. Neither model has been found to be complete, and thus a third model has emerged which describes stress as “the result of a mismatch between individuals’ perceptions of the demands of the task and their perceptions of the resources for coping with them” [5]. Staal also explored the similarities and differences between stress and mental workload. He described that, while increased mental workload may be a stressor (i.e., a stimulus that induces a physiological stress response), it does not always induce stress. A critical factor is whether the mental workload increases beyond an individual’s comfort level or ability. Therefore, whether a certain mental workload level induces stress is often related to the level of experience an individual has in executing a particular task. Furthermore, stressful stimuli and states need not be accompanied by high mental workload [5]. 

More specifically, research on non-invasively quantifying human stress has emphasized the use of several different physiological measurements such as blood volume pulse, galvanic skin response, pupil diameter, and skin temperature, as well as clinical-grade scalp electroencephalography (EEG) [6,7]. Variations in these signals are well-known indicators of sympathetic nervous system responses to cognitive stress [8]. The related field of mental workload analysis has produced a large literature specifically focused on classification problems [9,10,11], and new approaches for improving classifier performance are the aim of many recent studies in this area. Roy et al. [12] compared mental workload classification performance using spectral features extracted from raw EEG to performance using task-independent spatially-filtered event-related potentials (ERPs) elicited in a reportedly minimally intrusive manner. They relied upon a visual ERP evoked by an exogenous probe stimulus (an image of a shape) that is distinct from the experimental stimulus (an image of a number) they used to cue recall in a number-list memorization task. They concluded that ERPs yielded a better performance (91% accuracy) when compared with spectral markers (60%). This is an encouraging result, but one whose practical application is limited to scenarios in which the introduction of an exogenous visual probe is both feasible and non-disruptive.

In addition to studying EEG feature selection, researchers have also addressed the unique challenges associated with classifying workload in minimally constrained scenarios. For example, Zarjam et al. [13] aimed to assess working memory load in a manner that did not require active user participation, in real-time, ruling out the inclusion of ERPs in their experimental design. They extracted entropy, energy, and wavelet coefficient-based features from EEG signals, and achieved high classification accuracy, including subject-independent multi-channel classification that was stable over time. Schultze-Kraft et al. [14] explored unsupervised learning approaches for handling missing or noisy data labels. They leveraged cross-frequency power correlations to classify different states of workload with a mean accuracy of 82%, noting that classifier performance was improved when EEG data were augmented with peripheral physiological measures.

Raduntz et al. [15] sought to overcome the need to retrain a classifier upon the introduction of new subjects and/or tasks. They used dual-frequency head maps on at least 54 subjects and three different workload conditions to train a static support-vector machine (SVM) classifier using frequency spectrum-based features. They demonstrated that a general increase in frontal theta power and a decrease in parietal alpha power differentiated between workload conditions. The performance in classifying workload states of new subjects and/or tasks was sufficient without retraining.

Whether successes in classifying mental workload using EEG translate naturally to discriminating differences in cognitive stress states remains an open question. Addressing the difference between workload and stress, Gaillard [5] concluded that high workload may but does not always induce stress. Staal [4] provided evidence that the Stroop test, in particular, has been successfully employed as a stimulus that puts subjects under cognitive stress, allowing researchers to measure the downstream effects on cognition. 

Some past studies have induced stress using Stroop or Stroop-type (hereafter, both referred to as “Stroop” for simplicity) tests and then quantified it using non-EEG physiological measurements [1]. Others have employed Stroop tasks while recording EEG but primarily analyzed the data to draw conclusions about the effects of stress on cognition rather than using EEG features to distinguish different stress states. Astolfi et al. [16] used a Stroop task as a stimulus to look for an indirect measurement of cortical connectivity. Schack et al. [17] analyzed real-time EEG coherence in a Stroop paradigm to observe the evolution cortical connectivity measures. Atchley et al. [18] used only the non-stress inducing stimulus of the Stroop task to induce boredom, facilitating the exploration of EEG for features indicative of mind-wandering. Coelli et al. [19] used Stroop stimuli in assessing user engagement as an indirect measure of cognitive processing.

Other related work has used physiological measurements in attempting to distinguish among emotional states such as confusion, anger, hate or more generally among negative, neutral and positive emotional states. Zhou et al. [20] successfully detected a state of confusion from EEG features. Another study recorded both EEG and other physiological signals while subjects were asked to remember three different past emotional events [21]. This study found EEG signals superior (79% accuracy) to peripheral signals (53%) for classification of the induced emotional states. Finally, Hosseini [22] used EEG to successfully classify two different image-induced emotional states. 

**Figure 1:**
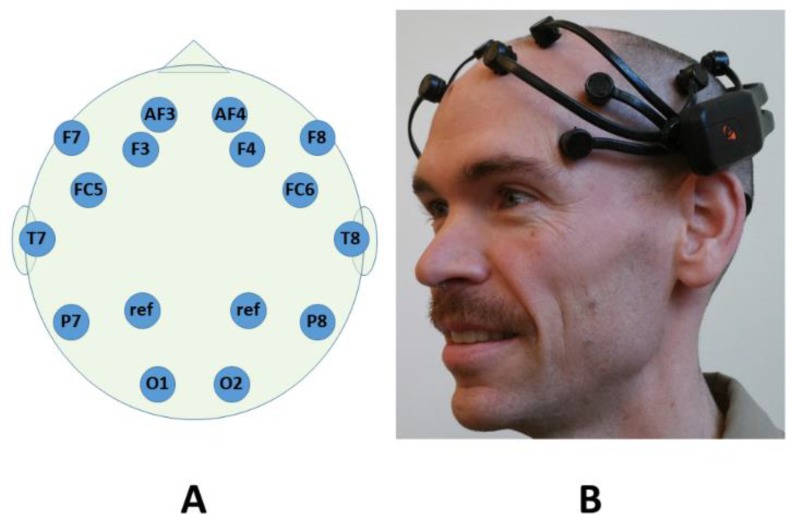
Scalp electrode locations (**A**) covered by the Emotiv Epoc system; and the Emotiv Epoc system as it would be worn by a subject (**B**). The electrode labeling convention follows the Modified Combinatorial 10–20 Standard [24].

Recent advancements in technology have made neural signal acquisition and processing more affordable and portable, and opened up the possibility of characterizing the human stress response in mobile environments outside the laboratory or clinic. One example of such a system is the Emotiv Epoc neuroheadset (Emotiv, San Francisco, CA, USA), shown in Figure 1. The headset was originally targeted at gaming, artistic, market research, and low degree-of-freedom brain–computer interface applications (e.g., wheelchair control), but it can be combined with open source software (e.g., BCI2000 [23]) for use in research applications. The Epoc headset is at least an order of magnitude less expensive than a clinical-grade EEG system. It also does not require the use of a conductive gel, although it does require applying a sterile saline solution to the electrodes.

The Emotiv Epoc system consists of three major components: a Bluetooth wireless headset with 14 electrodes; a USB wireless receiver; and signal acquisition and processing software. The Epoc continuously records 14 channels of EEG at a sampling rate of 128 Hz. It uses hardware notch filters at 50 and 60 Hz and a digital highpass filter with a cutoff frequency of 0.1 Hz. Figure 1 shows the relative locations on the scalp of the system’s 14 active and 2 reference electrodes, which are named and positioned according to the Modified Combinatorial 10–20 Standard [24]. 

Some studies [25,26] have attempted to replicate and extend the work in [3] using Stroop stimuli in conjunction with the Emotiv Epoc headset, also with promising results. Directly comparing the performance metrics reported in these studies with either previous work or the current study is difficult, however, as they employ test conditions (e.g., resting vs. incongruent) expected to generate greater between-level contrast, and use either subject-tailored methods of stress induction or subject self-assessment of stress level in labeling their data and stratifying it prior to statistical analysis. Ekanayake [27] demonstrated that the performance of the Emotiv Epoc system on a P300 task was competitive with clinical-grade systems. Knoll et al. [28] used the Epoc as a low-cost EEG solution for distinguishing between different levels of cognitive workload during reading tasks. Even more recently, Wang et al. [29] recorded EEG to classify n-back memory tasks of varying difficulty in real-time. They successfully extracted frequency spectral features and used SVM classification to achieve 80–100% performance accuracies. They concluded that the Emotiv Epoc system is a low-cost mobile EEG data acquisition system that is effective for their application. 

In the present study, we combined the Emotiv Epoc system with BCI2000, a brain–computer interfacing software package that we used for signal acquisition and stimulus delivery. Custom MATLAB (MathWorks, Natick, MA, USA) scripts were developed for all analyses. Our experimental design and a subset of results reported in Section 3.1 and 3.2 were originally described in [3]. These initial results are expanded upon in this work.

## 2. Materials and Methods

This within-subjects experimental study was conducted at the US Naval Academy, USA. It was conducted in conformance with the Declaration of Helsinki. The protocol was approved by the Institutional Review Board of the US Naval Academy (Human Research Protection Program approval #2012.0024-IR-EP7-A) and informed consent was obtained from study participants. 

### 2.1. Experimental Task 

We used a Stroop color–word interference test [2] to elicit mild stress responses in 18 college-aged male subjects. The test involves displaying the text string of a color–word, such as “blue," and rendering it in a font color that either matches or does not match its definition. Subjects are tasked with indicating the font color of the word via keypress (Sichel and Chandler [2] used a verbal response). Incongruent Stroop stimuli such as the latter are well established as cognitive stressors that induce emotional responses and heightened levels of physiological reactivity [1].

### 2.2. Data Collection

Subjects took a computer-based version of the Stroop test while their neural signals were recorded by the Epoc EEG headset and their reaction times (i.e., time between the initial appearance of the color–word on the screen and the initiation of their response) were logged. The computerized Stroop test was developed using BCI2000’s “Stimulus Presentation Module,” which is designed to present a sequence of visual and/or auditory stimuli to a subject [7]. Visual stimuli (color–words) were created in Microsoft Paint and imported into the Stimulus Presentation Module. Each subject participated in 10 experimental sessions: 5 “congruent” sessions, in which the font color and word matched, and 5 “incongruent” sessions, in which they did not. A session consisted of 24 trials, each comprised of a color–word stimulus lasting 1 s coupled with a blank screen period (i.e., the inter-stimulus interval) of session type-dependent duration (see Table 1). Because previous work indicated that adding pacing to the Stroop test might intensify physiological responses [1], inter-stimulus intervals were set such that all trials expired within 1 s of the disappearance of the color–word stimulus, and intervals between incongruent stimuli were varied uniformly randomly between 0.5 and 1 s. If subjects did not respond to each stimulus within the allotted time, the screen automatically updated to the next trial. Table 1 gives parameter values used for stimulus delivery in the congruent and incongruent sessions. 

**Figure 2:**
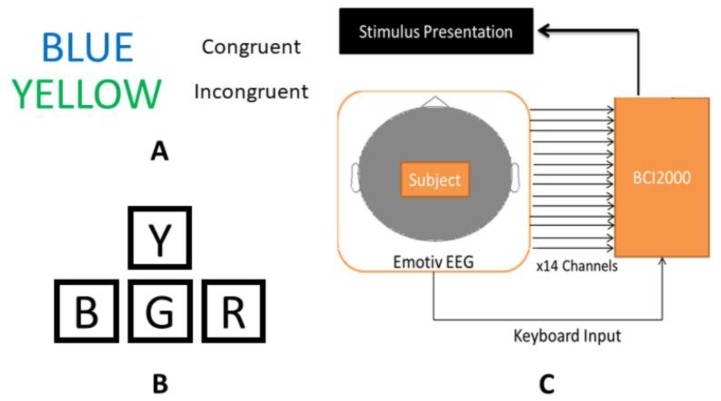
Experimental design components: examples of congruent and incongruent Stroop color–word stimuli (**A**); schematic of modified arrow keys on a standard keyboard, used to record subject responses to Stroop stimuli (**B**); and diagram of the hardware and software configuration used for data collection (**C**).

Before experimental sessions began, subjects were seated in front of the computer monitor and instructed that they would be required to respond to visual stimuli displayed on the monitor by typing the first letter of the font color of the displayed word. Subjects then participated in 2–3 practice sessions consisting of 24 congruent words to gain familiarity with the computer–subject interface and ask any questions before beginning the experiment as described above. Examples of congruent and incongruent color–word stimuli are shown in Figure 2, along with a schematic of the keyboard layout used for subject input―modified arrow keys on a standard keyboard―and a diagram of the hardware and software systems used for data acquisition.

#### 2.2.1. Pre-Processing

Raw EEG data were detrended by subtracting the least-squares line of best fit and passed through a bandpass filter network of Chebychev Type II filters with approximately 1 Hz transition widths, 1 dB of allowable passband ripple, and 80 dB of stopband attenuation, consisting of three bands of interest within the frequency interval 4–30 Hz: the theta band (θ; 4­­–8 Hz), the alpha band (α; 8–13 Hz) and the beta band (β; 13–30 Hz). Data were filtered in forward and reverse to achieve zero phase response. (To minimize any potential impacts of on- and offset transients, we also tested similarly specified equiripple FIR filters, shifting the outputs to compensate for group delay, with negligible impact on our results.) We chose to do our initial analysis in these three bands because previous work has indicated a high correlation between cognitive stress or workload and relative power in the theta, alpha, and beta bands [9,10,25,26,30]. In addition, restricting analysis to these bands reduced the influence of high frequency noise and low frequency artifacts such as EOG (electrooculogram) and EMG (electromyogram) on the EEG signal. 

One sensor, AF3 (left frontal), was eliminated from analysis after it was determined to be faulty (pairwise correlations >0.99 between all possible combinations of the theta, alpha, and beta RMS features computed on this electrode suggested it was recording only non-physiologic noise). Data from the remaining 13 sensors were retained for analysis.

#### 2.2.2. Windowing and Feature-Extraction

For each experimental session, the bandpass filtered data were divided into non-overlapping segments, one corresponding to each of the presented words. Each segment was 1 s in duration beginning at the initiation of word presentation. This method is similar to a feature extraction approach used in a study in which event-related potentials were detected in the context of a Stroop task [7]. For the alpha, beta, and theta bands, the root mean squared value (RMS) of the signal over the entire 1-s window was computed for each of the 24 total segments in each experimental session. The three RMS values were then concatenated for each segment, forming a 24 × 3 feature matrix for each session. 

#### 2.2.3. Individual Electrode Classification

After extracting the theta, alpha, and beta RMS features, we compared the accuracy of three supervised learning techniques in discriminating incongruent from congruent segments based upon these features. The first method was logistic regression [31], in which a linear class boundary was learned by assuming sigmoid models for the posterior probabilities of the two classes (congruent and incongruent) and finding the model parameters that maximize the probability of the observed data. The second method was quadratic discriminant analysis (QDA) [31], a more flexible method capable of finding a curved class boundary (a quadratic surface). In this method, test observations are assigned to the class with largest posterior probability assuming multivariate Gaussian class-conditional densities, and model parameters are estimated via maximum likelihood. The third method of classification was the k-Nearest Neighbor (k-NN) algorithm [31], a highly flexible nonlinear method that assigns class labels to test points according to a plurality vote among the closest training points in feature space. For k-NN, we used Euclidean distance as the measure of closeness and 3 as the number of neighbors (k = 3). These three classifiers were selected because they are representative state-of-the practice methods [31] that span the spectrum of model flexibility from linear methods with potentially high bias but low variance to highly non-linear methods with potentially low bias but high variance. The overall goal was to generally identify the model flexibility-level with the most favorable bias-variance tradeoff for these data, as measured by cross-validation-estimated test set performance (see below).

**Figure 3:**
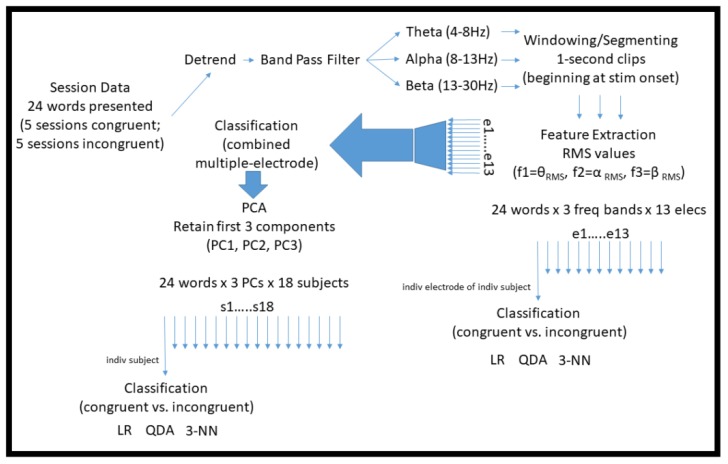
Diagram illustrating the main computational techniques used to pre-process and classify the raw EEG data in the initial analysis methodology described in Section 2.2.1, Section 2.2.2, Section 2.2.3, Section 2.2.4, Section 2.2.5 and Section 2.2.6.

Since each experimental session was homogenous (i.e., contained either all congruent or all incongruent words), to minimize potential confounds associated with adaptation or fatigue across sessions, five separate training datasets were formed by combining ordinally corresponding pairs of congruent and incongruent sessions. An individual classifier was learned for each subject–electrode–session pair combination. Generalization performance was assessed using leave-one-out cross-validation (LOOCV) [31], and summarized for each subject–electrode by taking the median accuracy over the five pairs of sessions. (To be concise, hereafter when discussing the initial analysis methodology described in Section 2.2.1, Section 2.2.2, Section 2.2.3, Section 2.2.4, Section 2.2.5 and Section 2.2.6, we refer to this median accuracy simply as the “performance” or “accuracy” of the classifier.) Inside the cross-validation loop, training features were normalized by subtracting the mean and dividing by the standard deviation of each feature. Test points were accordingly transformed using the training mean and standard deviation. Figure 3 summarizes the main computational techniques used to process the data in this initial analysis methodology. 

To examine the potential influence of time-trends in the EEG signal unrelated to the heightened stress induced by color–word incongruence, we also built null classifiers that attempted to discriminate early (the first 12) from late (the last 12) segments in each congruent session. We then compared the performance of these null classifiers with our stress-state classifiers, as well as with chance classifiers formed by randomly permuting the congruent and incongruent labels.

#### 2.2.4. Fused Feature Classification

After considering the classification accuracy at each electrode individually, a natural question was whether performance across subjects could be improved by using information from all electrodes simultaneously. To test this idea, we formed 39-element feature vectors by fusing (concatenating) the theta, alpha, and beta features from each of the 13 electrodes. To allow a more direct comparison with the unfused feature case, we then used Principal Components Analysis (PCA) [31] to reduce the dimensionality of these new feature vectors to 3, retaining on average 85.3% of the total data variance in doing so. Reducing the size of the feature space in this way has the side benefit of lessening computation time. It may also mitigate adverse impacts on performance associated with attempting to learn decision boundaries in high-dimensional spaces given our relatively small training set sizes (n = 47 for LOOCV). However, we made no explicit attempt to optimize the number of features used.

We then trained and tested logistic regression, QDA, and 3-NN classifiers on the data using leave-one-out cross-validation, performing the PCA step inside the cross-validation loop and mapping test-points to the PCA space of the training data, paralleling the procedures used for the individual electrode data. 

#### 2.2.5. Timing and Persistence of the Stress Response

We also investigated whether, during incongruent experimental sessions, the stress response was globally heightened throughout the session or specifically locked to the delivery of the incongruent stimuli. To test this, we extracted the same theta, alpha, and beta band features from the data, only now locking analysis windows to randomly generated (i.e., spurious) stimulus delivery times rather than the true delivery times of the color–word stimuli presented in each session. We then compared classification results for this randomized case with those obtained using the true stimulus-delivery times.

#### 2.2.6. Feature Analysis

Finally, we were interested in whether particular electrode–RMS feature combinations were generally useful across subjects for discriminating between congruent and incongruent trials. Toward this end, we compared the loadings of the first principal component among subjects. Since the principal components are linear combinations of the original features, examining the relative magnitudes of the elements of the first principal component loading vector―whose direction in feature space is that along which the variance of the projected data is maximal―gives insight into which electrode–RMS feature combinations may play strong (and weak) roles in distinguishing congruent from incongruent trials. 

#### 2.2.7. Alternative Analysis Framework

As both a test of the robustness and an extension of the analytical procedures just described, we also reconstituted every major analysis detailed above in Section 2 using an alternative framework that allowed us to enlarge both our feature and training sets. In detail, we aggregated experimental sessions instead of considering them in temporally corresponding pairs, in this case excluding the final congruent and incongruent sessions (approximately the last two of about ten recorded minutes for each subject) to mitigate the effects of task fatigue and adaptation. We then computed additional time-domain features that are both common in the EEG signal processing literature and have shown promise in our previous work in quantifying cognitive workload: EEG signal line length, number of peaks, and mean Teager energy [11]. Specifically, we computed the following five additional features for every 1-s EEG segment analyzed on each subject-channel: the respective mean theta-, alpha-, and beta-band Teager energy signals (three separate features); the number of positive-going peaks in the wideband (i.e., absent any bandpass filtering) EEG signal; and the line length of the wideband signal. To balance the computational burden in the face of this large increase in feature vector and training set size and the stability of our test set performance estimates, we used 10-fold [31] rather than leave-one-out cross-validation. 

Under this alternative analysis framework, the size of our training sets nearly quadrupled, from 47 to ~172 observations on every subject-channel, and the size of our feature vectors expanded by ~167%. For example, our fused feature analysis now considered 104 features prior to PCA, rather than the previous 39 features. This alternative procedure has both potential costs and benefits relative to the procedure described in Section 2.2.1, Section 2.2.2, Section 2.2.3, Section 2.2.4, Section 2.2.5 and Section 2.2.6. On the one hand, the increase in the heterogeneity of the dataset brought about by aggregating across sessions might be expected to drive classification performance downward. On the other hand, the increase in training and feature set sizes might be expected to improve it.

## 3. Results

### 3.1. Reaction Time for the Stroop Task

**Figure 4:**
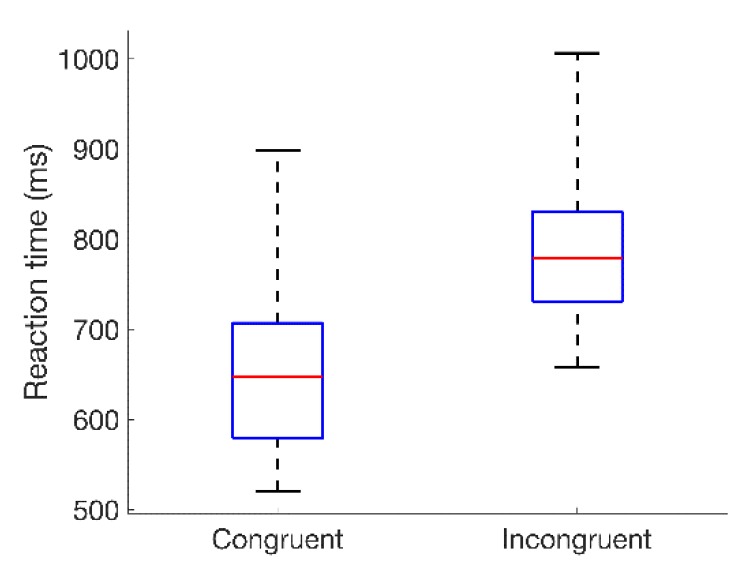
Box-and-whisker plots summarizing the distributions of reaction times for all 18 subjects to congruent and incongruent color–word stimuli. Values represented are median (across sessions) average reaction times relative to the onset of the color–word stimulus, with averages taken across the 24-word presentations in each session. Red lines indicate the medians of each distribution; blue boxes span the first to third quartiles; and whiskers extend to the extreme values.

The distributions of reaction times (RTs) for all 18 subjects are summarized for the congruent and incongruent conditions in Figure 4. A Wilcoxon signed-rank test [32] showed a statistically significant difference in the distributions of reaction times across subjects for the congruent and incongruent conditions (p < 0.001). This is consistent with the incongruent Stroop condition increasing cognitive stress, and in agreement with prior research [1].

### 3.2. Individual Electrode Classification

**Figure 5:**
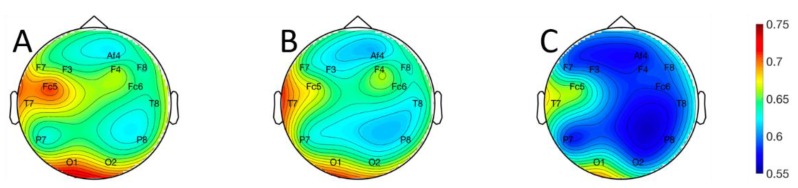
Topographical plots of classification performance averaged across all 18 subjects for the: (**A**) logistic regression; (**B**) quadratic discriminant analysis; and (**C**) three-nearest neighbor classifiers. Values represented by the color maps are the proportion of correctly labeled examples. Values at non-electrode locations were obtained via biharmonic spline interpolation [33]. The electrode labeling convention follows the Modified Combinatorial 10–20 Standard [24].

The logistic regression, QDA, and 3-NN classifiers were evaluated using leave-one-out cross-validation, with classification accuracy as the performance metric. Topographical plots of classification accuracy averaged across subjects are shown for the logistic regression (Figure 5A), QDA (Figure 5B), and 3-NN (Figure 5C) classifiers in Figure 5. Continuous representations were obtained by using the classification accuracies at each electrode location, which are represented exactly, to interpolate between electrodes [33]. Data at the discrete electrode locations are represented in tabular form in Table 2. 

**Figure 6:**
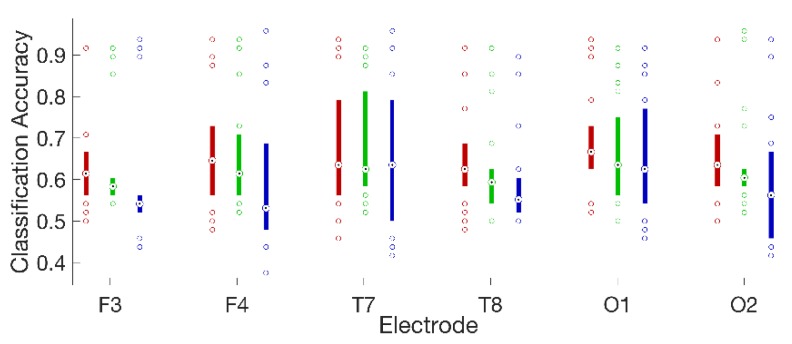
Box plots summarizing the distributions of classification accuracies across all 18 subjects for the logistic regression (red); quadratic discriminant analysis (green); and three-nearest neighbor classifiers (blue), on three laterally symmetric pairs of frontal (F3 and F4), temporal (T7 and T8), and occipital (O1 and O2) electrodes. Medians are shown as black dots inside colored circles and boxes span the interquartile range. Values falling outside the interquartile range are plotted as unfilled circles to explicitly show the number of subjects at the extremes of each distribution.

For 16 of 18 subjects, on all electrodes, the logistic regression classifier had the highest median accuracy, followed by the QDA classifier, and then the 3-NN classifier. The performance differences among classifiers were statistically significant for four electrodes: F3, F4, F7, and O2 (Friedman’s test [32]; Bonferroni corrected [32] p < 0.05 in all cases). It is possible that a larger number of subjects would have permitted detection of significant effects at more electrode locations. At the same time, the performance differences we observed among classifiers at statistically significant electrodes were modest (roughly 2–5%). This suggests that, while an increase in statistical power brought about by enlarging the number of subjects might yield some benefit, it could also give rise to easily misinterpreted results with statistical but limited practical significance. The highest classification accuracy for a single subject was 97.9% on left fronto-central electrode Fc5, and the highest average accuracies across subjects were 70.7% on electrode Fc5 and 68.1% on left occipital electrode O1. Classifier performance varied widely across subjects. The distributions of accuracies across subjects for a frontal, temporal, and occipital sensor on each hemisphere are plotted in Figure 6 for each of the three classifiers. 

To investigate whether our results might be influenced by EEG time-trends unrelated to the heightened stress induced by color–word incongruence, we built null classifiers that attempted to discriminate early (the first 12) from late (the last 12) segments in each congruent session. As expected, the logistic regression classifier had significantly better performance than its corresponding early-versus-late segment null classifier, as did the 3-NN classifier (Wilcoxon signed-rank test on the distributions over electrodes of subject-mean classifier performance; Bonferroni corrected p < 0.001 in both cases). However, this was not the case for the QDA classifier. Furthermore, all three of the early-versus-late segment null classifiers outperformed chance classifiers built by randomly permuting the class labels (Wilcoxon signed-rank test on the distributions over electrodes of subject-mean classifier performance; Bonferroni corrected p < 0.01 in all three cases). Taken together, these results suggest that our stress-state classifiers are in part leveraging time-trends in the EEG signal unrelated to the heightened stress induced by color–word incongruence. It is difficult to say conclusively whether this reflects an artifact of the experimental paradigm or a more interesting secondary stress response associated with within-session fatigue. 

### 3.3. Fused Feature Classification

**Figure 7:**
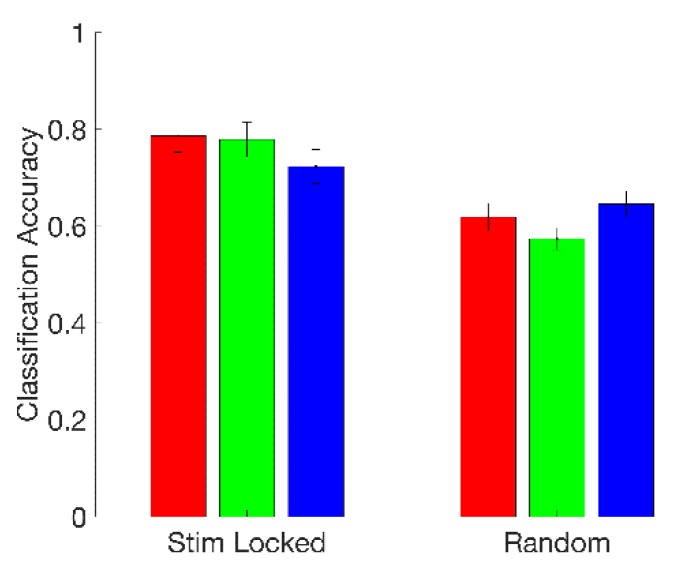
Fused-feature classification mean accuracy across subjects for logistic regression (red), quadratic discriminant analysis (green), and 3-NN (blue), when 1-s feature extraction windows were triggered by the onset of the color–word stimulus (“Stim Locked”) and when 1-s feature extraction windows were taken at random times (“Random”). Error bars are two standard errors in length.

We found that fusing features across electrodes gave a substantial boost in classification performance, even after reducing the dimensionality of the newly formed 39-element fused-feature vectors to three dimensions via PCA. With the previous single-electrode approach, the best mean classification accuracy we found was 70.7%. Using fused features, we found a mean accuracy of 78.7% across subjects for the top-performing classifier (logistic regression), with 5/18 subjects having classification accuracies greater than 90%. Logistic regression and QDA (77.9%) had statistically indistinguishable performance, while both were superior to 3-NN (72.3%) (Friedman’s test, p < 0.001; post-hoc Wilcoxon signed-rank test; Bonferroni-corrected p < 0.05 in both cases). These results are summarized on the left-hand side (“Stim Locked”) of Figure 7. 

Paralleling the analyses for the individual electrode classifiers in Section 3.2, we compared our fused-feature stress state classifiers to null classifiers formed by training on early (first 12) versus late (last 12) congruent segments, as well as to chance classifiers built by randomly permuting the congruent and incongruent class labels. General conclusions were analogous (although, in this case, since electrode data were fused, classifier accuracy distributions were compared across subjects rather than electrodes). 

**Figure 8:**
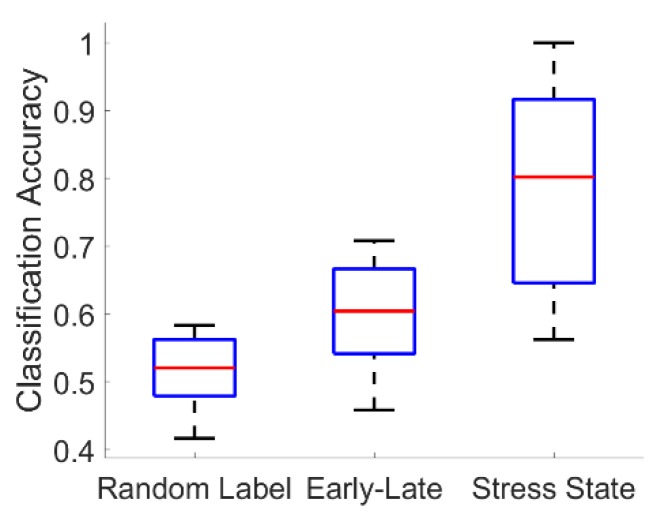
Summary of representative fused-feature classification results. Boxplots show the distribution of classifier accuracies across all 18 subjects for a chance logistic regression classifier formed by randomly permuting the congruent and incongruent labels (“Random Label”); a null logistic regression classifier formed by training to discriminate early versus late segments in congruent experimental sessions (“Early-Late”); and the actual logistic regression stress-state classifier trained to discriminate congruent from incongruent segments (“Stress State”).

For the fused-feature classifiers, all stress-state classifiers substantially outperformed their corresponding null early-versus-late segment classifiers (Wilcoxon signed-rank test; Bonferroni-corrected p < 0.001 in all three cases). At the same time, all but one (3-NN) early-versus-late segment classifier statistically significantly outperformed its corresponding chance classifier formed by randomly permuting the congruent and incongruent labels (Wilcoxon signed-rank test; Bonferroni-corrected p < 0.05 for logistic regression and QDA). Representative results are shown for the logistic regression classifier in Figure 8.

### 3.4. Timing and Persistence of the Stress Response

We found that the timing of stress stimuli is important. The logistic regression, QDA, and 3-NN classifiers all performed better when 1-s feature extraction windows were locked to the onsets of color–word stimuli than when analysis windows were triggered at random times. This suggests that experimental sessions produce stress responses directly connected to or amplified by individual stimuli, as opposed to the sessions being only globally more or less stressful depending on the nature (incongruent or congruent) of the color–words being presented. At the same time, the results for the random analysis window experiment are significantly better than chance (Wilcoxon signed-rank test against classifiers formed by randomly computing class labels; Bonferroni-corrected p < 0.05 for all classifiers), which suggests that there could additionally be a more global component to the stress response, perhaps related, for example, to the subject’s perceived success rate at the task, the inter-stimulus interval durations, or variations in attentiveness. Our conclusions on this point are limited by the results of our early-versus late segment classification experiments, as well as the fact each of our random analysis windows, or some portion thereof, had a relatively high probability of falling in close vicinity to an actual stimulus due to the nature of the experimental design (see parameters in Table 1). The results of the timing experiment are summarized in Figure 7.

### 3.5. Feature Importance Analysis

**Figure 9:**
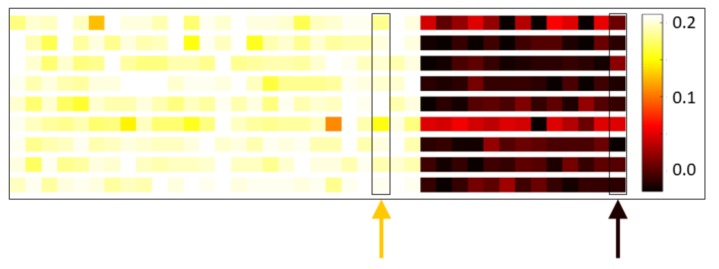
First principal component loadings. Visualization of the average first principal component loadings for nine randomly selected subjects (one subject per row). Each row consists of 39 colored panels, one for each of the 39 electrode–RMS feature combinations used for classification. The panels are colored in proportion to the contribution of their corresponding feature to the first principal component; they are ordered in three blocks of 13 corresponding to the 13 analyzed electrodes, with the leftmost block representing the theta RMS feature, the middle block representing the alpha RMS feature, and the rightmost block representing the beta RMS feature. Within each block, the electrode ordering is as follows: F7, F3, Fc5, T7, P7, O1, O2, P8, T8, Fc6, F4, F8, and Af4. For example, the leftmost panel in all rows represents θ RMS on electrode F7 and the rightmost panel β RMS on Af4. Lighter color panels indicate electrode–RMS feature combinations with stronger positive weightings. The orange arrow indicates one particular combination that has relatively high-magnitude weight across subjects; the black arrow indicates one with relatively low-magnitude weight.

We found electrode–RMS feature combinations whose contributions to the first principal component were relatively consistent across subjects. This interestingly suggests there are specific combinations of electrode locations and RMS features that perform well at discriminating stress state in a general fashion. Figure 9 gives “heat map” representations of the average (across combined congruent–incongruent session pairs) first principal component loadings for nine randomly selected subjects. Each row corresponds to a subject and consists of 39 colored panels, one for each of the 39 electrode–RMS feature combinations used for classification. The panels are colored in proportion to the contribution of their corresponding feature to the first principal component; they are ordered in three blocks of 13, corresponding to the 13 analyzed channels, with the leftmost block representing the theta RMS feature, the middle block representing the alpha RMS feature, and the rightmost block representing the beta RMS feature. Immediately apparent from this ordering is the fact that all electrode–beta RMS feature combinations are contributing relatively weakly to the first principal component across subjects. It is also worth noting that many of the rows display a similar color spectrum, indicating in a broader sense that similar electrode–RMS feature combinations are making similar relative contributions to the first principal component across subjects. The orange arrow in Figure 9 shows one particular combination that contributes relatively strongly across subjects (electrode F4, alpha RMS feature) and the black arrow a combination that contributes relatively weakly across subjects (electrode Af4, beta RMS feature). 

**Figure 10:**
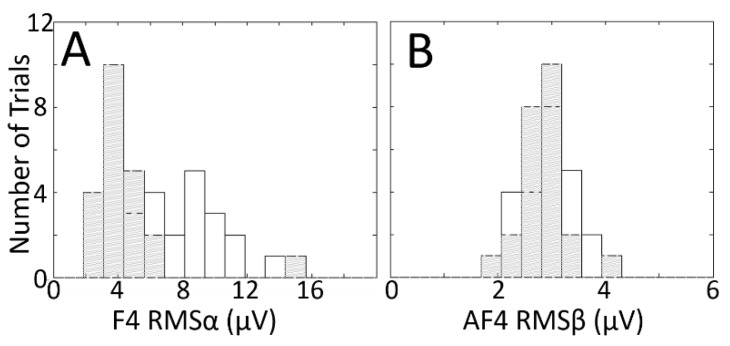
Histograms showing the distributions of RMS voltage across trials (color–word stimulus presentations) in one congruent (grey hatched) and one incongruent (white) experimental session pair, for one electrode–RMS feature combination that shows relatively good class separation (**A**) and one that shows relatively poor class separation (**B**). The electrode–RMS feature combinations illustrated in (A,B) correspond to the orange and black arrows, respectively, in Figure 9. Data in (A,B) are from two different subjects. Outliers in both panels are trimmed to avoid scaling issues that hamper visualization.

Histograms for these two combinations, shown in Figure 10, further illustrate the point. In Figure 10A, the histograms for the congruent (grey hatched) and incongruent (white) conditions show separation, supporting the utility of the F4–RMSα combination for classification; in Figure 10B, the histograms are overlapping, suggesting that the Af4–RMSβ is a poor combination for discerning the two classes. 

### 3.6. Alternative Analysis Framework

We found that the costs and benefits of the alternative analysis procedure described in Section 2.2.7. were largely counterbalancing. Results were broadly consistent in trend between the two analysis approaches, with the alternative framework yielding an approximately identical best performance for the individual electrode analysis (70.8%) and a modest performance boost (81.1%) in the fused feature analysis. There were also some expected differences: for example, a statistically insignificant shift in the electrode showing the highest performance (T7 for the second analysis, as compared with Fc5 in the first). 

In general, the performance of 3-NN improved relative to the other two classifiers, likely owing to a decrease in model variance accompanying the increased training set size. Still, logistic regression remained the strongest performing classifier overall, with the highest accuracy on a majority of electrodes for 13 of 18 subjects in the individual electrode analysis, and the highest performing (including one tie) classifier for 11 of 18 subjects in the fused feature analysis. The most notably different result between the analysis frameworks was for the timing experiment described in Section 2.2.5: the differences in classification performance between the random and stimulus-locked conditions visible in Figure 7 were not significant in the alternative analysis framework. An effective signal-to-noise ratio enhancement brought about by increasing the number of features with discriminatory power could account for this finding; increasing the number of features need not have the same (or even any) impact on classification performance for both conditions. 

Figures analogous to Figure 4, Figure 5, Figure 6, Figure 7, Figure 8, Figure 9 and Figure 10, showing the main results of the alternative analysis framework, are provided as Supplementary Materials (Figures S1–S7). Overall, the results of the two different analysis procedures were remarkably similar, strengthening our conclusions.

## 4. Discussion

Our results demonstrate that, under controlled conditions, a portable, low cost-EEG headset can be used to achieve good accuracy in distinguishing two different cognitive stress states on a single-trial basis, using spectral features extracted from individual electrodes (70.7% mean across subjects for electrode Fc5, for example). Furthermore, performance can be substantially enhanced (78.7%) by taking advantage of the information on all electrodes simultaneously. We found accuracies greater than 90% for 5/18 subjects using a logistic regression classifier with such a fused feature approach. Notably, these results were obtained on a single trial basis using extremely small training sets: only 47 words when corresponding congruent and incongruent sessions were combined. When we substantially increased both the training set and the number of features used in classification, we saw a modest boost in performance, to 81.1% accuracy in the fused feature case—in spite of the increase in training set heterogeneity incurred by aggregating across experimental sessions. The exclusion of the final congruent and incongruent sessions to mitigate the effects of task fatigue and adaptation in this alternative analysis framework may have contributed to the observed performance boost. Our results also suggest that the stress-state classifiers are to some degree leveraging time-trends in the EEG signal unrelated to the heightened stress induced by color–word incongruence. If these time-trends are unrelated to stress altogether, their presence could mean our results are optimistically biased. However, they may also reflect a more interesting secondary stress response associated with within-session fatigue. The inability to completely disentangle this issue is a limitation of our study design, which used trial-homogenous sessions and did not independently measure another physiological variable, such as galvanic skin response, that is correlated with stress-response. It would be interesting to explore alternative experimental paradigms that would include the measurement of other physiological variables and also might prospectively attempt to control for fatigue and/or measure it as part of the procedure.

The differences we observed between stress states with a low-cost, consumer-grade device in a non-clinical setting are consistent with previous work done in the field of stress quantification in clinical settings [30], and our classification performance was reasonably comparable to, though lower than, related work done with devices substantially more costly. For example, Schultze-Kraft et al. [14] reported a classification accuracy of 90.4% when using a feature extraction approach similar to ours in a cognitive workload study using the actiCAP System (BrainProducts GmbH, Germany), a clinical-grade device that is an order of magnitude more expensive than the Emotiv Epoc.

In a comparative study, the Emotiv Epoc headset was shown to be influenced by movement artifacts and yield EEG signals with signal-to-noise ratios that are poorer than a medical-grade system [34]. In less motion-constrained studies than ours that may be severely impacted by eye and other movement artifacts, some error sources can potentially be mitigated by applying established artifact rejection techniques [35,36], although this can be challenging without the flexibility to place additional reference signal electrodes offered by medical-grade devices. The analysis done here is not conclusive with respect to the Emotiv Epoc’s ability to have application outside of controlled laboratory environments. It also cannot address potential gender differences in neurophysiological responses to stress, as our subject pool was comprised of only one gender.

The present study sought to provide a baseline analysis of two cognitive stress states, as induced by incongruent and congruent color–word stimuli in a Stroop test paradigm, using a portable, wireless, low-cost, consumer-grade EEG headset. Future research in this area will be aimed at studying whether these positive results can be translated to non-laboratory environments and mobile environments, and whether they can be used to establish a continuous measure of cognitive stress that can be tracked in real time. 

## Figures and Tables

**Table 1:** Parameter values for presentation of Stroop color–word stimuli in experimental sessions.

Session Type	Number of Stimuli	Stimulus Duration (s)	Inter-Stimulus Interval (s) (Offset-to-Onset)
Congruent	24	1	0.5
Incongruent	24	1	0.5–1 (uniform random)

**Table 2:** Electrode-specific classification performance averaged across all 18 subjects for the logistic regression, quadratic discriminant analysis, and three-nearest neighbor classifiers.

Location	Logistic Regression	QDA	3-NN
F7	65.74%	64.12%	59.49%
F3	64.81%	62.50%	59.03%
Fc5	70.72%	67.01%	65.62%
T7	68.29%	68.63%	66.09%
P7	63.19%	63.66%	58.10%
O1	69.44%	67.13%	65.39%
O2	66.44%	64.58%	59.38%
P8	63.31%	62.73%	58.80%
T8	64.12%	62.50%	59.37%
Fc6	64.47%	64.12%	58.56%
F4	65.86%	65.62%	58.68%
F8	63.66%	63.19%	59.72%
Af4	62.38%	61.46%	57.64%

## Supplementary Materials

Supplementary figures are available online at http://www.mdpi.com/1424-8220/19/3/499/s1.
