# Supplementary material for: Single-Trial Cognitive Stress Classification Using Portable Wireless Electroencephalography
Source: Sensors (Basel). 2019 Jan 25;19(3):499. doi: 10.3390/s19030499 (PMC6387350; doi:10.3390/s19030499)
Supplement: Supplementary file 1 [file sensors-19-00499-s001.zip › sensors-407833_Second_revision_supplementary_material.pdf]

Supplementary Material

What follows are analogous versions of each figure from the Results section of the main manuscript (Figures 4-10), obtained when the alternative analysis framework described in Sections 2.3.7. and 3.6. is used. Results were generally consistent between the two analysis methodologies we investigated, with key differences highlighted in the main manuscript.

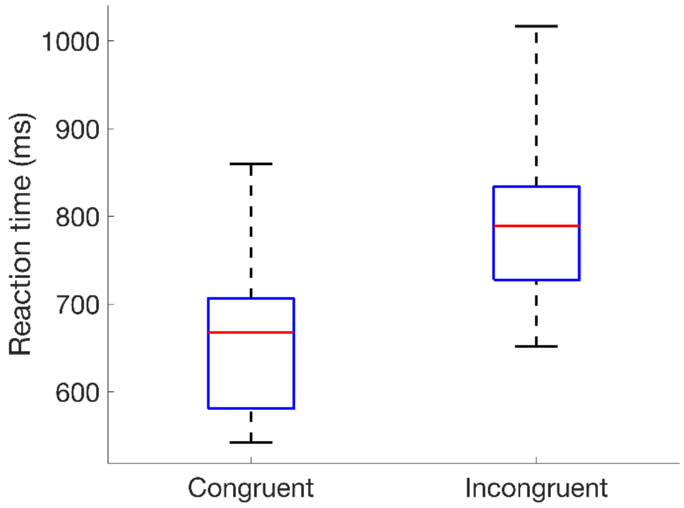

Figure S1. Box-and-whisker plots summarizing the distributions of reaction times for all 18 subjects to congruent and incongruent color-word stimuli. Values represented are average reaction times relative to the onset of the color-word stimulus, with averages taken across the 96 word presentations of each type (congruent and incongruent) analyzed for each subject. Red lines indicate the medians of each distribution; blue boxes span the first to third quartiles; and whiskers extend to the extreme values.

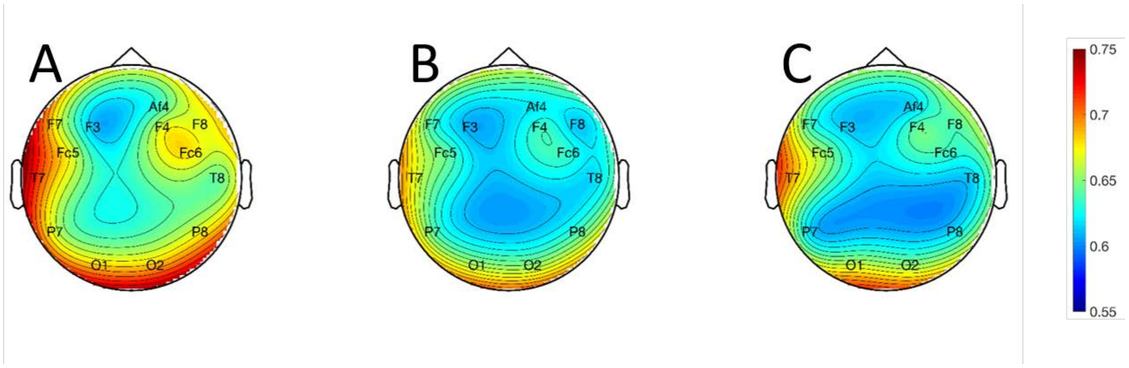

Figure S2. Topographical plots of classification performance averaged across all 18 subjects for the (**A**) logistic regression; (**B**) quadratic discriminant analysis; and (**C**) 3-nearest neighbor classifiers. Values represented by the color maps are proportion of correctly labeled examples. Values at non-electrode locations are obtained via biharmonic spline interpolation [33]. The electrode labeling convention follows the Modified Combinatorial 10-20 Standard [23].

Table S1. Electrode-specific classification performance averaged across all 18 subjects for the logistic regression, quadratic discriminant analysis, and 3-nearest neighbor classifiers.

Location	Logistic Regression	QDA	3-NN
F7	66.98%	63.19%	63.25%
F3	60.97%	60.68%	61.23%
Fc5	67.36%	64.35%	65.51%
T7	70.83%	66.52%	68.52%
P7	66.23%	64.09%	61.40%
O1	68.49%	65.83%	66.67%
O2	69.16%	65.62%	65.54%
P8	67.62%	64.06%	62.27%
T8	64.29%	61.75%	61.60%
Fc6	67.82%	63.25%	63.40%
F4	67.19%	63.54%	64.41%
F8	66.78%	61.17%	63.83%
Af4	63.51%	62.04%	61.69%

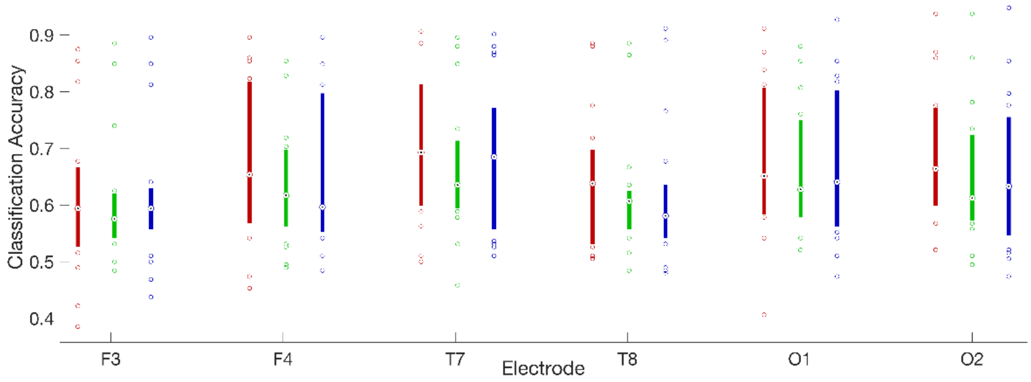

Figure S3. Box plots summarizing the distributions of classification accuracies across all 18 subjects for the logistic regression (red); quadratic discriminant analysis (green); and 3-nearest neighbor classifiers (blue), on three laterally symmetric pairs of frontal (F3 and F4); temporal (T7 and T8); and occipital (O1 and O2) electrodes. Medians are shown as black dots inside colored circles and boxes span the interquartile range. Values falling outside the interquartile range are plotted as unfilled circles to explicitly show the number of subjects at the extremes of each distribution.

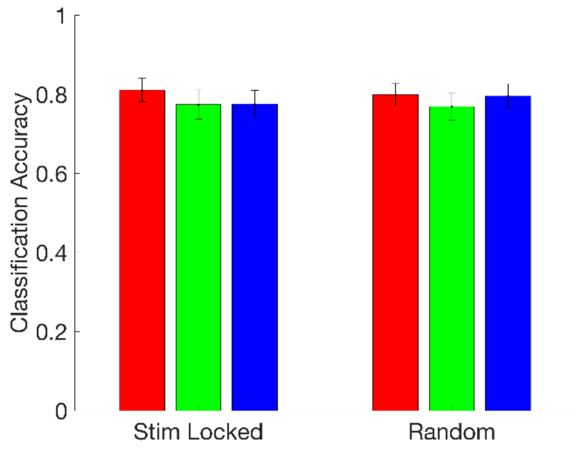

Figure S4. Fused-feature classification mean accuracy across subjects for logistic regression (red), quadratic discriminant analysis (green), and 3-NN (blue), when 1-second feature extraction windows were triggered by the onset of the color-word stimulus ("Stim Locked") and when 1-second feature extraction windows were taken at random times ("Random"). Error bars are two standard errors in length.

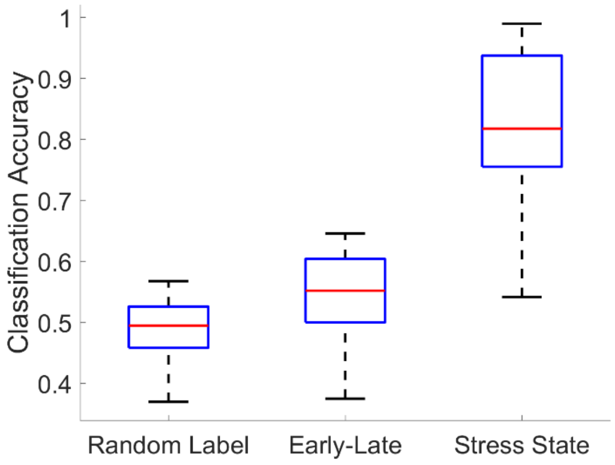

Figure S5. Summary of representative fused-feature classification results. Boxplots show the distribution of classifier accuracies across all 18 subjects for a chance logistic regression classifier formed by randomly permuting the congruent and incongruent labels ("Random Label"); a null logistic regression classifier formed by training to discriminate early versus late segments in congruent experimental trials ("Early-Late"); and the actual logistic regression stress-state classifier trained to discriminate congruent from incongruent segments ("Stress State").

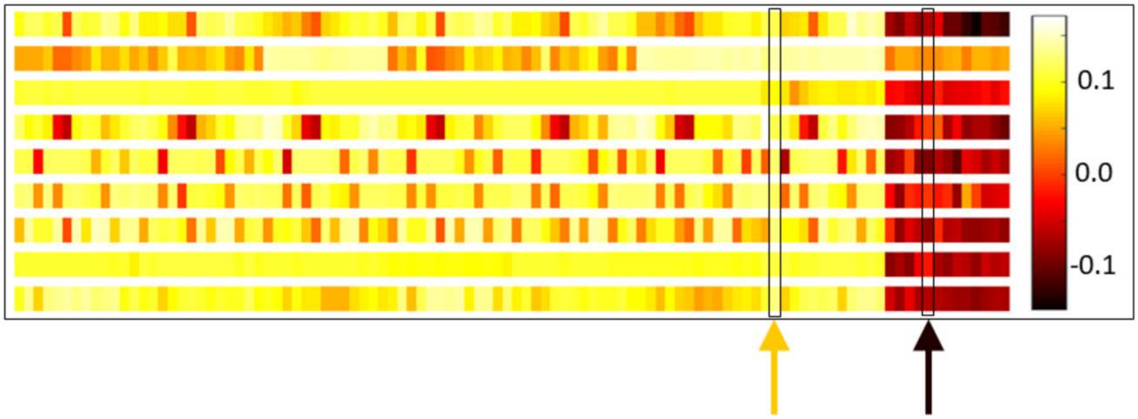

Figure S6. First principal component loadings. Visualization of the first principal component loadings for 9 subjects (the set-complement of the randomly selected subjects from Figure 9; one subject per row). Each row consists of 104 colored panels, one for each of the 104 electrode-feature combinations considered in classification. The panels are colored in proportion to the contribution of their corresponding feature to the first principal component; they are ordered in 8 blocks of 13 corresponding to the 13 analyzed electrodes, with block ordering as follows, from left to right: θ RMS, α RMS, β RMS, θ MTE, α MTE, β MTE, LL, NP. Within each block, the electrode ordering is as follows: F7, F3, Fc5, T7, P7, O1, O2, P8, T8, Fc6, F4, F8, Af4. For example, the leftmost panel in all rows represents the principal component loading corresponding to θ RMS on electrode F7 and the rightmost panel NP on Af4. Lighter colored panels indicate electrode-feature combinations with higher positive weightings. The orange arrow indicates one particular combination that has relatively high-magnitude weight across subjects; the black arrow indicates one with relatively low-magnitude weight. (RMS = Root mean square; MTE = Mean Teager energy; LL = Line length; NP = Number of peaks).

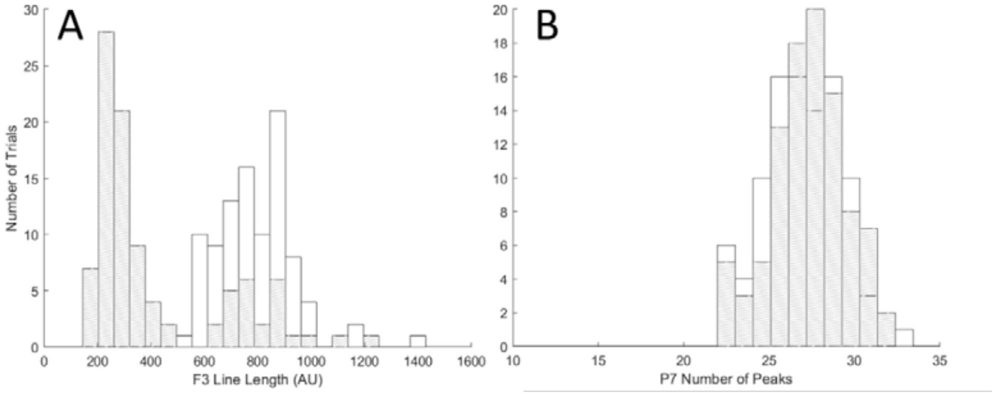

Figure S7. Histograms showing the distributions of two different electrode-feature combinations: F3 Line Length (A) and P7 Number of Peaks (B) used in the fused-feature analysis described in main manuscript sections 2.3.4. and 3.3. Each panel represents the corresponding electrode-feature combination values for all analyzed congruent (grey hatched) and incongruent (white) trials (color-word presentations) in a single subject, for one electrode-feature combination that yields relatively good class separation (A) and one that yields relatively poor class separation (B). The electrode-feature combinations illustrated in panels A and B correspond to the orange and black arrows, respectively, in Figure S6. Data in panels A and B are from two different subjects.
