# Supplementary figures and images for: Single-Trial Cognitive Stress Classification Using Portable Wireless Electroencephalography
Source: Sensors (Basel). 2019 Jan 25;19(3):499. doi: 10.3390/s19030499 (PMC6387350; doi:10.3390/s19030499)

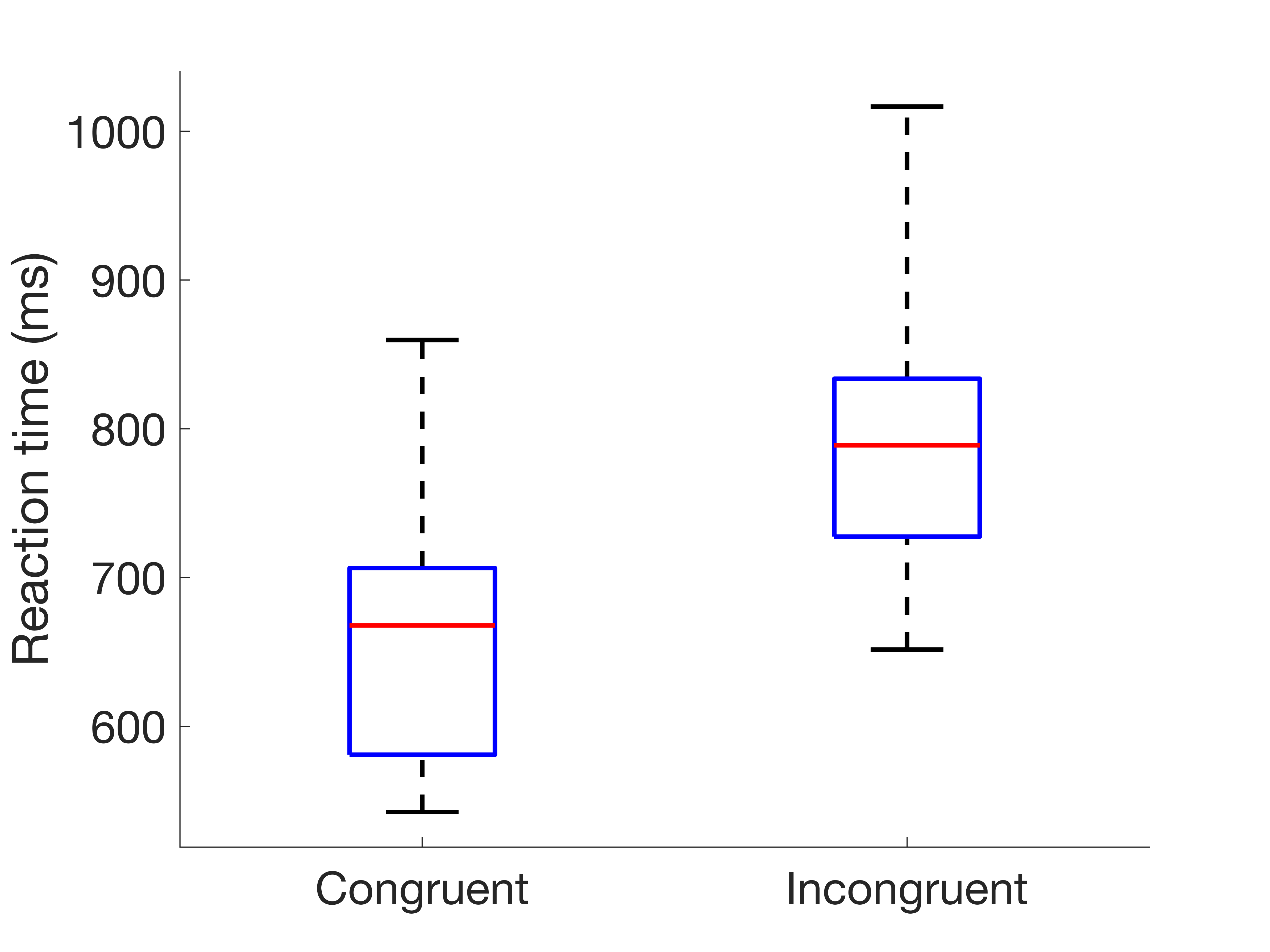

Supplement: Supplementary file 1 [file sensors-19-00499-s001.zip › FigureS1.tif]

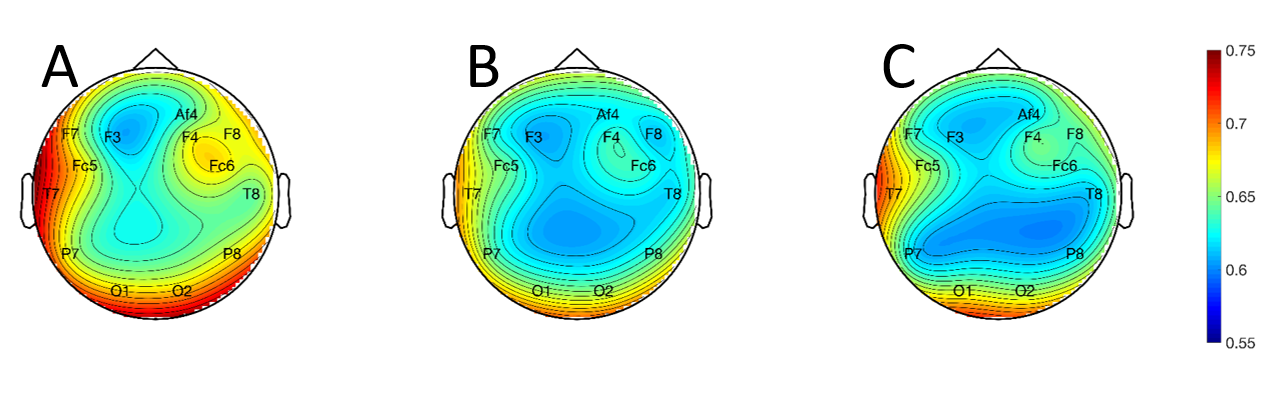

Supplement: Supplementary file 1 [file sensors-19-00499-s001.zip › FigureS2.tif]

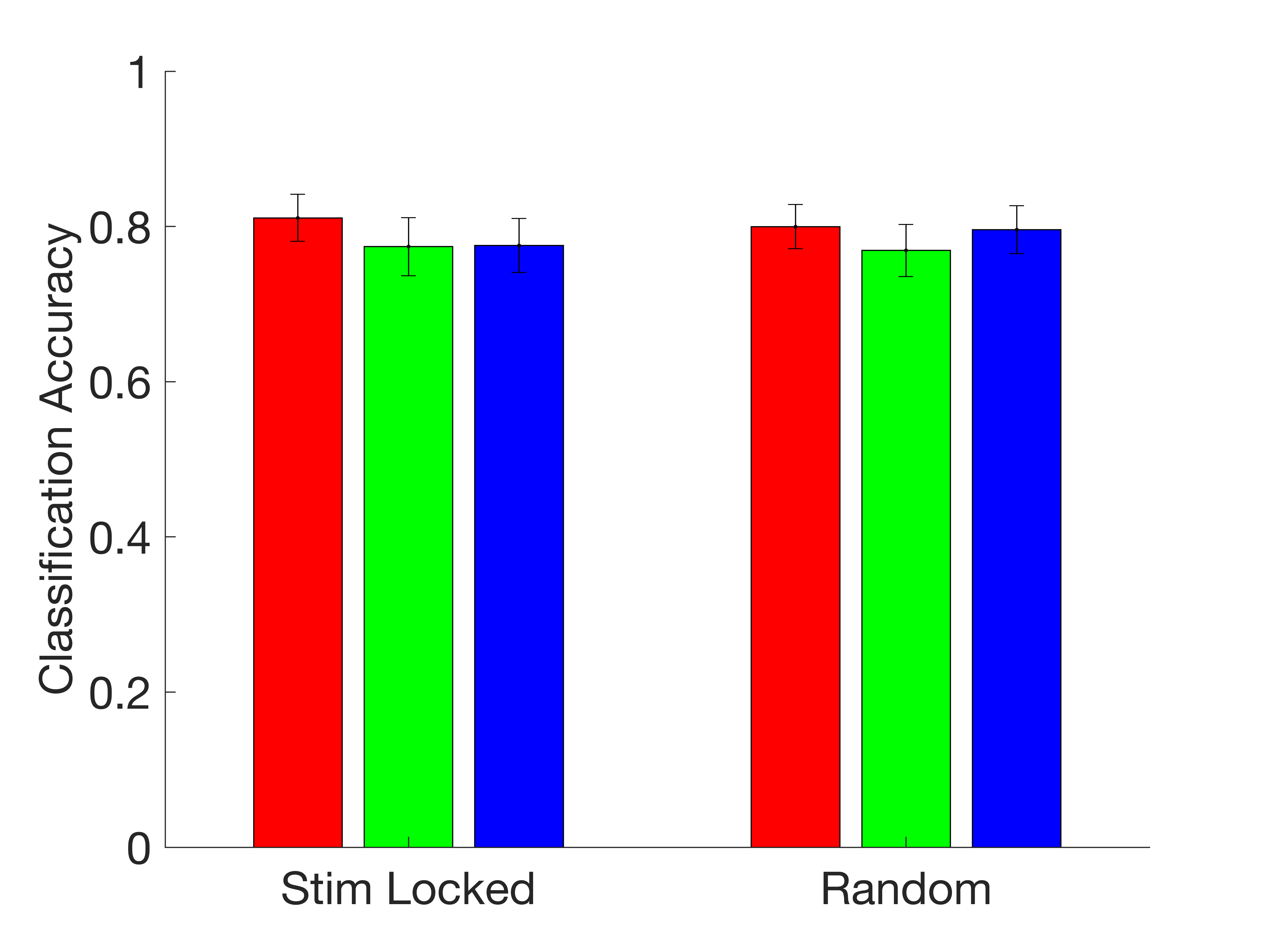

Supplement: Supplementary file 1 [file sensors-19-00499-s001.zip › FigureS4.tif]

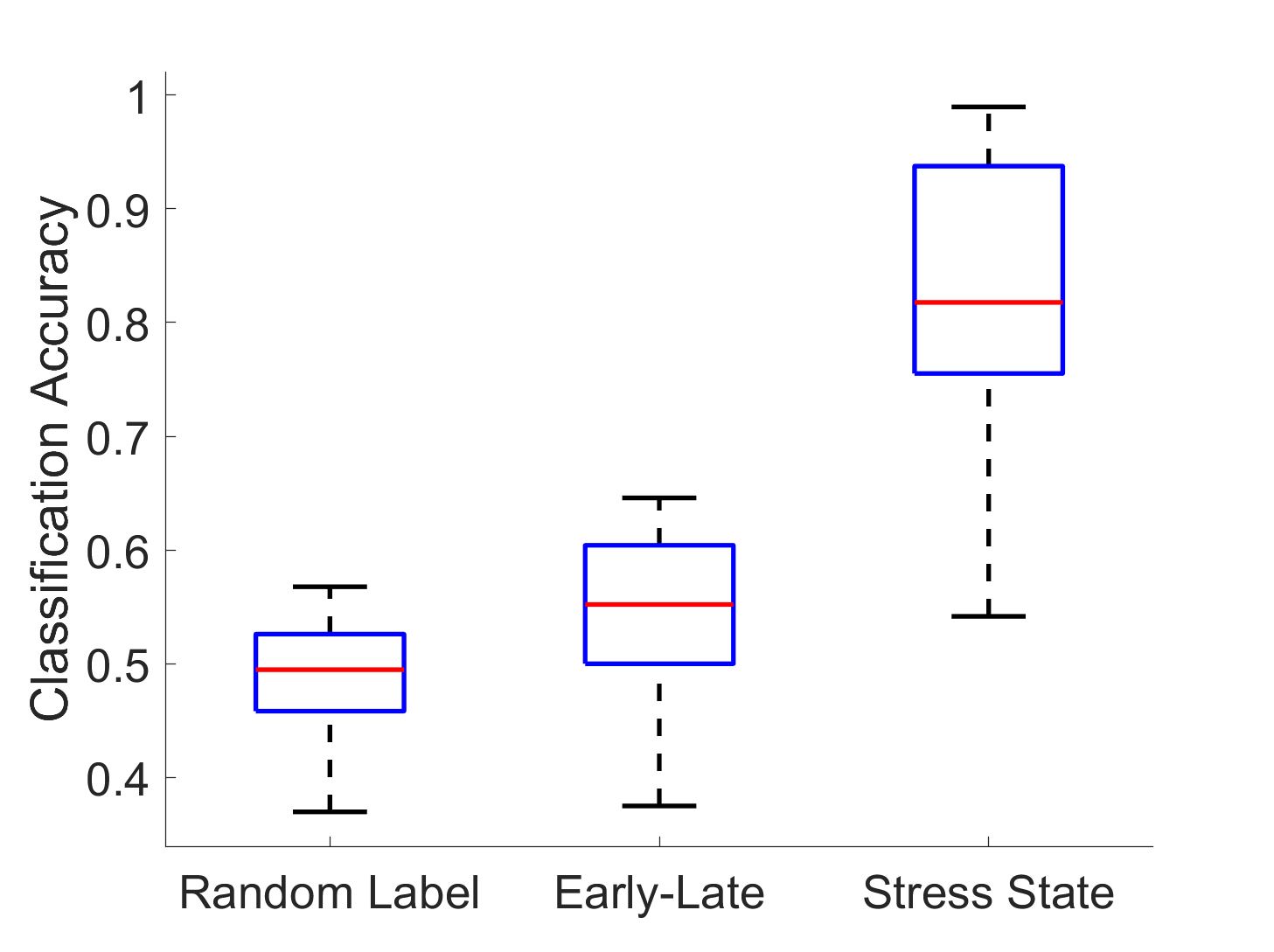

Supplement: Supplementary file 1 [file sensors-19-00499-s001.zip › FigureS5.tif]

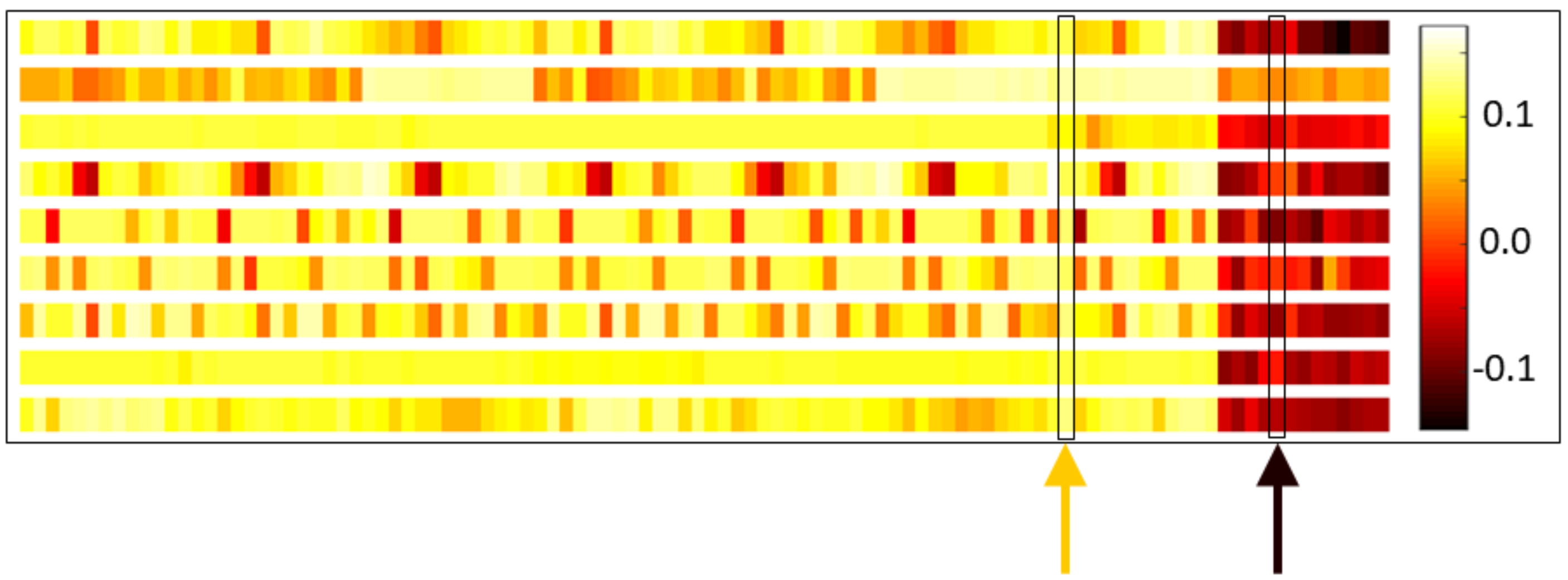

Supplement: Supplementary file 1 [file sensors-19-00499-s001.zip › FigureS6.tif]

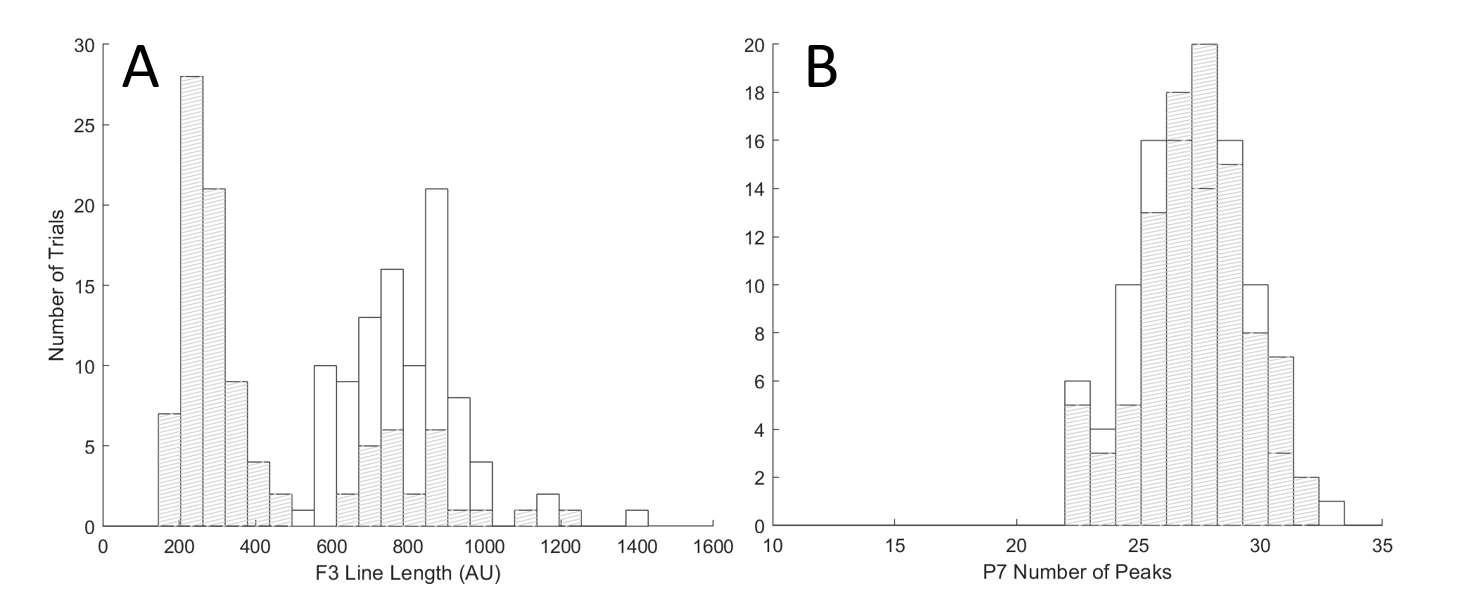

Supplement: Supplementary file 1 [file sensors-19-00499-s001.zip › FigureS7.tif]

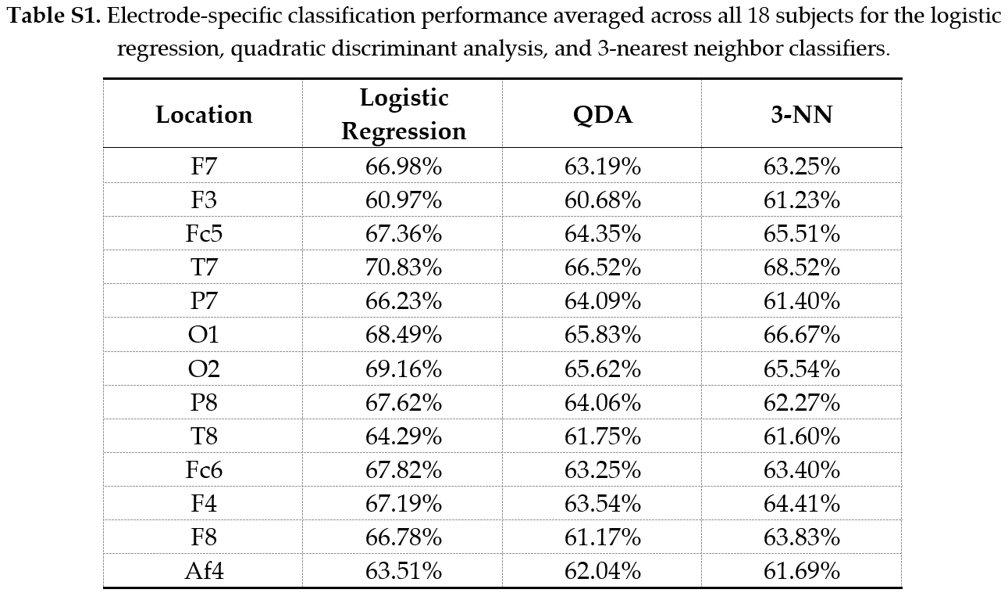

Supplement: Supplementary file 1 [file sensors-19-00499-s001.zip › TableS1.tif]
